# Calcitriol Inhibits Viability and Proliferation in Human Malignant Pleural Mesothelioma Cells

**DOI:** 10.3389/fendo.2020.559586

**Published:** 2020-10-08

**Authors:** Iacopo Gesmundo, Francesca Silvagno, Dana Banfi, Valentina Monica, Alessandro Fanciulli, Giacomo Gamba, Noemi Congiusta, Roberta Libener, Chiara Riganti, Ezio Ghigo, Riccarda Granata

**Affiliations:** ^1^Division of Endocrinology, Diabetes and Metabolism, Department of Medical Sciences, University of Turin, Turin, Italy; ^2^Department of Oncology, University of Turin, Turin, Italy; ^3^Candiolo Cancer Institute-Fondazione del Piemonte per l'Oncologia (FPO) Istituto di Ricovero e Cura a Carattere Scientifico (IRCCS), Candiolo, Italy; ^4^Pathology Unit, SS. Antonio e Biagio General Hospital, Alessandria, Italy

**Keywords:** calcitriol, cell proliferation, cell survival, malignant pleural mesothelioma, mitochondria

## Abstract

Malignant pleural mesothelioma (MPM) is a rare and aggressive tumor, often associated with exposure to asbestos and characterized by poor prognosis and limited treatment options. The biologically active form of vitamin D, calcitriol, exerts anticancer effects in many cell types, both alone and in combination with chemotherapy drugs, through binding to vitamin D receptor (VDR); however, the role of calcitriol in MPM is still unknown. This study aimed to determine the potential antitumor role of calcitriol in MPM. The results showed that calcitriol reduces cell viability and proliferation in human MPM cells lines, which express both cytoplasmic and nuclear VDR; furthermore, calcitriol potentiated the inhibitory activity of the chemotherapy drug PEM. These effects were paralleled by cell cycle arrest and inhibition in expression of c-Myc and cyclins involved in cell cycle progression. Exposure of MPM cells to calcitriol also produced an alteration in mitochondrial function and inhibition in the expression of respiratory chain complex subunits. Finally, the inhibitory effects of calcitriol were also observed on viability of human primary MPM cells. Collectively, these results indicate a novel anticancer role for calcitriol in MPM, suggesting potential for vitamin D derivatives, alone or in combination with chemotherapy, in the treatment of this malignancy.

## Introduction

Malignant pleural mesothelioma (MPM) is a rare and aggressive tumor with a dismal prognosis, that occurs because of malignant transformation of pleural mesothelial cells. The principal cause of MPM development is exposure to asbestos, although different minerals, such as erionite and radiation therapy in patients treated for other cancers, have been also implicated (1). Exposure to asbestos generates a chronic inflammatory state, with activation of macrophages, production of reactive oxygen species and mediators of inflammation, such as cyclooxygenases, cytokines and growth factors, all promoting tumor formation (2). Pathologically, some MPM have an epithelial morphology, the most frequent and less malignant variant, others sarcomatoid, with the worst prognosis, while a mixed biphasic population comprising both histologic subtypes also exists (2, 3). The first-line treatment for inoperable patients with MPM consists in a combination of cisplatin and the antifolate pemetrexed (PEM), which, however, increase overall survival only of 9–12 months, therefore, novel therapeutic strategies are urgently needed (4).

The active form of vitamin D, the steroid hormone 1α,25-dihydroxyvitamin D_3_ or calcitriol, regulates many biological processes, mostly mediated by the nuclear vitamin D receptor (VDR), which acts through both genomic, and non-genomic mechanisms (5). Calcitriol has an essential role in calcium homeostasis and also modulates biological functions, including cell growth, wound healing, neuromuscular, and immune functions. In addition, calcitriol shows potential anticancer activity by regulating signaling pathways involved in cell proliferation, differentiation, apoptosis, inflammation, invasion, angiogenesis, and metastasis (6, 7). Indeed, studies have demonstrated the antiproliferative and pro-differentiating effects of calcitriol *in vitro*, in several cancer cell models. Moreover, calcitriol and its analogs, alone or in combination with anticancer drugs, showed the ability to delay initiation and inhibit tumor progression *in vivo*, in animal models of cancer (5–7). Clinical trials have been also undertaken in different cancers; however, the responses were modest, likely because the doses of the compounds were too conservative, the studies inappropriately designed and conducted in patients with far-advanced disease (5, 7–9).

The resistance to the antiproliferative effects of the hormone is due to many causes, some linked to a defective expression or function of VDR or aberrant expression of vitamin D-metabolizing enzymes, others because of epigenetic mechanisms that may disrupt VDR signaling (6). Indeed, the presence of VDR in cancer cells is essential for the activity of calcitriol and high VDR expression has been associated with improved prognosis and reduced risk of death from cancer (5, 6).

VDR is present mainly in metabolic tissues, but also in almost all human cell types (10); furthermore, VDR expression is low in normal cells, increases in malignant cells and is reduced with tumor growth (5, 11). VDR is also expressed in human and rodent peritoneal mesothelial cells and human bronchial epithelial cells (12–15); however, the presence of VDR in pleural mesothelial cells has yet to be demonstrated. Studies have recently indicated that calcitriol reduces fibrosis and prevents epithelial-mesenchymal transition in human peritoneal mesothelial cells *in vitro* (12, 14), while vitamin D analogs reduce peritoneal fibrosis *in vivo* (15) through antinflammatory mechanisms. In addition to its nuclear localization, VDR has been recently localized in mitochondria and calcitriol was found to suppress mitochondrial respiration in cancer cell lines, keratinocytes and adipocytes, affecting both cell growth and differentiation, as well as lipid metabolism (16–19).

Only one study examined the role of vitamin D in mesothelioma to date (20). The Authors reported that dietary supplementation with cholecalciferol (vitamin D3) in transgenic mice exposed to asbestos did not reduce the incidence or severity of peritoneal mesothelioma. However, differently from most studies performed in human cancer xenografts (5, 8, 21), the effects of cholecalciferol were assessed in a mouse model of asbestos-induced mesothelioma and the direct action of cholecalciferol in mesothelioma cells was not examined.

Based on the abovementioned data and because of its antiproliferative, antinflammatory and antioxidant activities, we hypothesized that vitamin D would exert direct inhibitory effects in MPM cells. Thus, in the present study we examined the role of calcitriol, alone or in combination with chemotherapeutic drugs, on viability and proliferation of human MPM cell lines and primary cells obtained from patients with MPM; furthermore, we analyzed the mechanisms involved in these effects.

## Methods

### Reagents

1,25(OH)_2_D_3_ (Calcitriol), Pemetrexed, 2,5-diphenyl tetrazolium bromide (MTT), Roswell Park Memorial Institute (RPMI)-1640 medium, Ham's F12 medium, fetal bovine serum (FBS), bovine serum albumin (BSA), penicillin, streptomycin, amphotericin B, L-glutamine, primers and cell culture reagents were from Sigma-Aldrich (Milan, Italy). RT-PCR and Real-Time PCR reagents were from Life Technologies, Inc. (Invitrogen, Milan, Italy).

### Cell Lines

The human biphasic MPM cell line MSTO-211H and the human mesothelial cell line MeT-5A were purchased from American Type Culture Collection (ATCC; Manassas, VA, USA). The human epithelioid MPM cell line REN was kindly provided by Prof. Giorgio Scagliotti (Department of Oncology, University of Turin, San Luigi Gonzaga Hospital, Orbassano, Turin, Italy), as described previously (22). Cells were maintained at 37°C in a 5% CO_2_ humidified atmosphere in RPMI-1640 with 10% FBS, 2 mM L-glutamine, penicillin (100 U/ml), streptomycin (100 μg/ml) and 250 ng/mL amphotericin B and used between passages 12 and 25.

### Isolation and Culture of Human Primary MPM Cells

Human Primary MPM cells (3 epithelioid MPM, 3 biphasic MPM, 3 sarcomatoid MPM) were isolated from diagnostic thoracoscopies of MPM patients, as previously described (22). Briefly, tissues were digested in medium containing 1 mg/ml collagenase and 0.2 mg/ml hyaluronidase for 1 h at 37°C. Cells were seeded in culture and used within passage 6. The study was approved by the Ethical Committees of the Biological Bank of Mesothelioma, SS. Antonio and Biagio General Hospital, Alessandria, Italy, and San Luigi Gonzaga Hospital, Orbassano, Turin, Italy (#9/11/2011; #126/2016). The patients provided their written informed consent to participate in this study. Primary MPM cells were grown in Ham's F12 medium with 10% of FBS (normal medium, NM). All culture mediums were supplemented with L-glutamine (2 mM), penicillin (100 U/ml), streptomycin (100 μg/ml), and 250 ng/mL amphotericin B. The cells were cultured at 37°C in a 5% CO_2_ humidified atmosphere.

### Cell Viability and Proliferation

Cells were seeded in 96-wells plates at the concentration of 2 × 10^3^ cells/well. After 48 h, cells were serum-starved for 12 h and incubated with the different stimuli for further 24 h or 72 h. Cell viability was assessed by 3-[4,5-dimethylthiazole-2-yl]-2,5-diphenyltetrazolium bromide (MTT) assay. Briefly, cells were incubated with 1 mg/ml of MTT for ~2 h, then the medium was removed, and formazan products solubilized with 100 μl dimethyl sulfoxide (DMSO). Cell proliferation was assessed using the 5-bromo-2-deoxyuridine (BrdU) incorporation enzyme-linked immunosorbent assay (ELISA) (Roche Diagnostic) as previously described (22). Absorbance was assessed by spectrophotometry at 570 nm for MTT and at 450 nm for BrdU, using LT-4000 microplate reader (Euroclone, Milan, Italy).

### Clonogenic Assay

Colony-forming capacity was analyzed by the clonogenic assay, as described previously (22). MSTO-211H REN and MeT-5A cells were seeded into 60 mm tissue culture plates, at a concentration of 1 × 10^3^ cells, and maintained in RPMI-1640 with 10% FBS for 10 days, in either presence or absence of 100 nM calcitriol. The cells were then fixed with methanol, colonies were stained with crystal violet (0.05%) and plates photographed using a digital camera (ChemiDoc XRS). Colonies were counted with ImageJ software (http://rsbweb.nih.gov/ij/).

### Western Blot Analysis

Protein extraction and Western blot analysis were performed as described previously (23). Total proteins were extracted using RIPA buffer (Sigma-Aldrich, Milan, Italy). Cytoplasmic and nuclear proteins were extracted using the Cytoplasmic and Nuclear Protein Extraction Kit (Immunological Sciences, Rome, Italy), following the manufacturer's instructions. Proteins (60 μg) were separated by 10 % sodium dodecyl sulfate—polyacrylamide gel electrophoresis, (SDS-PAGE), transferred to a nitrocellulose membrane and incubated overnight at 4°C with the specific Vitamin D3 Receptor antibody (dilution 1:1000, Cell Signaling, Beverly, MA, USA). Blots were reprobed with actin or PARP-1 (dilution 1:500, Santa Cruz Biotechnology, Dallas, USA) for protein normalization. Immunoreactive proteins were visualized using horseradish peroxidase-conjugated goat anti-mouse or goat anti-rabbit (1:4000) secondary antibodies (Southernbiotech, Birmingham, AL, USA) by enhanced chemiluminescence substrate (ECL) using ChemiDoc XRS (Bio-Rad, Milan, Italy); densitometric analysis was performed with Quantity One software (Bio-Rad, Milan, Italy).

### Real-Time PCR

Total RNA isolation and reverse transcription to cDNA (1 μg RNA) from MeT-5A, MSTO-211H and REN cells, treated with TRIzol reagent (Life Technologies, Milan, Italy), were performed as described previously (22). For real-time PCR, cDNAs were treated with DNA-free DNase (Life Technologies, Milan, Italy) and reaction performed with 50 ng cDNA, 100 nM of each primer and IQ-SYBR-green Mastermix (Bio-Rad) using the ABI-Prism 7,300 (Applied Biosystems, Milan Italy). The following primer pairs (designed with the Primer 3 Software, http://www.primer3.org/) were used: c-Myc, forward 5′- AGCGACTCTGAGGAGGAACA-3′, reverse 5′-CTCTGACCTTTTGCCAGGAG-3′ (NM_002467.5); cyclin-A, forward 5′-AATTGTGCCTTGCCTGAGTGA-3′, reverse 5′-AAGAACTGCAGGTGGCTCCAT-3′ (XM_011535295.2); cyclin D1, forward 5′-ATGTGTGCAGAAGGAGGTCC-3′, reverse 5′- CCTTCATCTTAGAGGCCACG-3′ (NM_053056.3); cyclin D2, forward 5′-TGCAGAAGGACATCCAACC-3′, reverse 5′-AGGAACATGCAGACAGCACC-3′(NM_001759.4); COX2, forward 5′-CGACTACGGCGGACTAATCT-3′, rev 5′-TCGATTGTCAACGTCAAGGA-3′ (EU835118.1); COX4, forward 5′-CGAGCAATTTCCACCTCTGT-3′, reverse 5′-GGTCAGCCGATCCATATAA-3′(NM_001861.6); 18S rRNA, forward 5′-CCCATTCGAACGTCTGCCCTATC-3′, reverse 5′-TGCTGCCTTCCTTGGATGTGGTA- 3′ (NR_146144.1), 18S rRNA was used as endogenous control. Relative quantification was performed using the comparative Ct (2^−ΔΔCt^) method.

### Cell Cycle Analysis

Cell cycle analysis was performed as described previously (24), using the Muse Cell Cycle Kit (Merck-Millipore, Burlington, MA, USA) according to the manufacturer's instructions. Briefly, 5 × 10^4^ cells were seeded in 60-mm dishes and after 48 h incubated with different concentrations of calcitriol in medium supplemented with 2.5% FBS for 24 h. Cells were then detached with phosphate buffered saline (PBS) 1X/ethylenediaminetetraacetic acid (EDTA) (5 mM), centrifuged (1,500 rpm, 5 min) and fixed with pre-cooled ethanol. The cells were then treated with Muse Cell Cycle Reagent for 30 min and analyzed with Muse Cell Analyzer Software (Merck-Millipore, Burlington, MA, USA).

### Measurement of Mitochondrial Membrane Potential (ΔΨm)

ΔΨm was assessed by cytofluorimetric analysis using the fluorescent probe JC-1 (5,5,6,6-tetrachloro-1,1,3,3-tetraethylbenzimidazole carbocyanide iodide JC-1 (Thermo Fisher Scientific, Waltham, MA USA), a cationic dye that shows mitochondrial polarization by shifting its fluorescence emission from green (530 nm) to red (590 nm). Consequently, the enhanced mitochondrial activity is indicated by an increase in the red/green fluorescence intensity ratio (FL2/FL1 channels of flow cytometer). The cells were treated with calcitriol for 24 h then harvested by trypsinization, washed in PBS (pH 7.4) and incubated with JC-1 (2 μg/ml) for 30 min at 37°C. The amount of JC-1 retained by 1 × 10^4^ cells per sample was measured at 530 nm (FL-1 green fluorescence) and 590 nm (FL-2 red fluorescence) using a flow cytometer FACSCalibur (BD biosciences, Franklin Lakes, NJ, USA) and analyzed with BD FACStation software (22, 25). ΔΨm was determined as FL2/FL1 ratio.

### Combination Studies

The synergistic effect of the drugs was performed according to the Chou-Talalay method of synergy quantitation using CompuSyn software (26). CI values were generated by performing cell viability assay (MTT) and computerized software data. CI = 1 indicates an additive effect between two drugs; a CI > 1 indicates antagonism, and a CI < 1 indicates a synergistic effect.

### Statistical Analysis

Results are presented as mean ± SEM. Significance was calculated by unpaired two-tailed Student's *t*-test or one-way ANOVA followed by followed by Dunnett's or Tukey's *post-hoc* test, as appropriate, using GraphPad Prism v.5 (GraphPad Software, SanDiego, CA, USA).

## Results

### Expression of Cytoplasmic and Nuclear VDR in MPM Cell Lines

**Figure 1:**
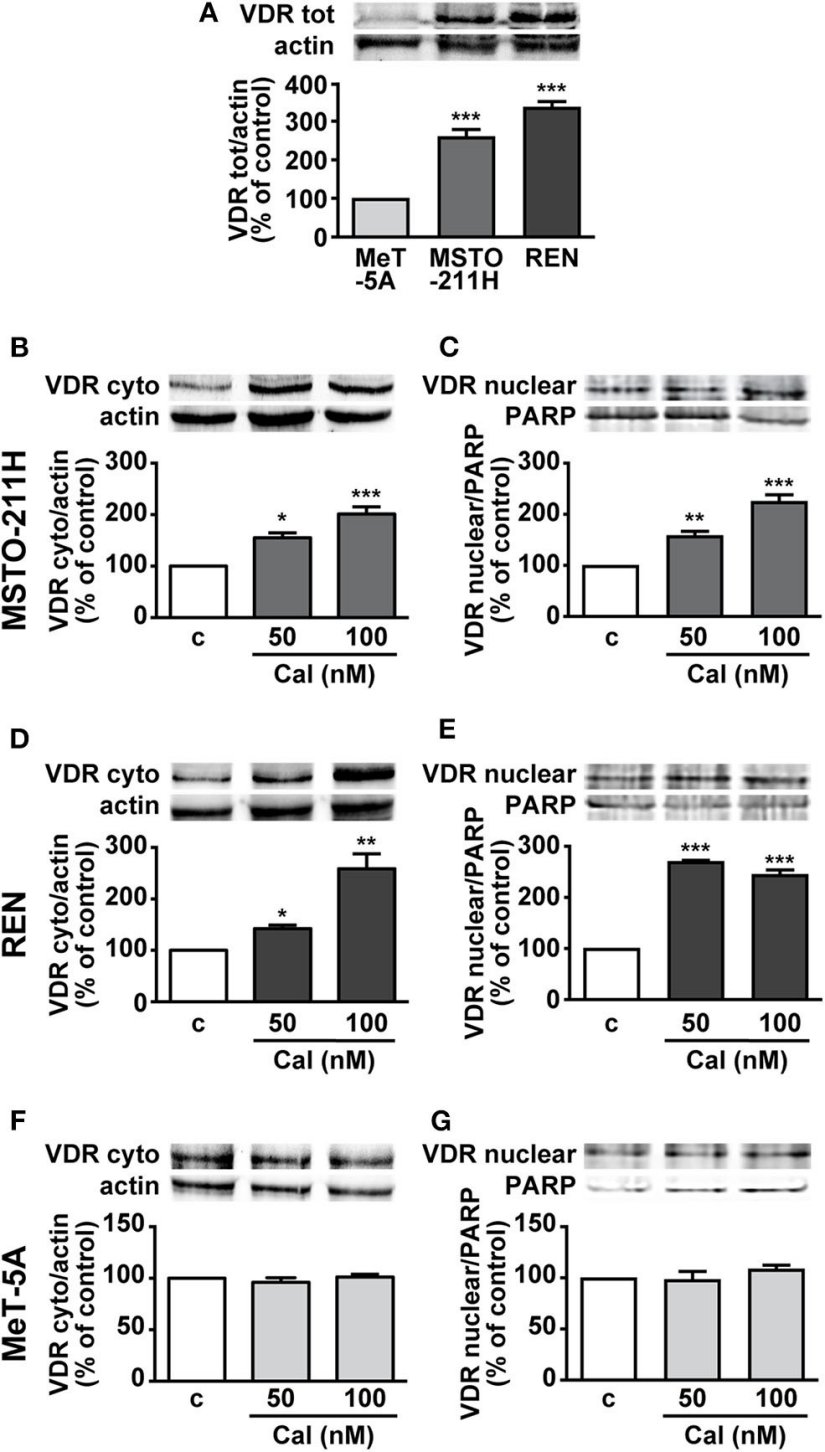
Expression of VDR in MPM cell lines and mesothelial cells. **(A)** Total VDR protein assessed by Western blot in MSTO-211H, REN and MeT-5A cells. Results, normalized to actin and expressed as percent of control are means ± SEM. ***P < 0.001 vs. MeT-5A; *n* = 3. Cytosolic and nuclear VDR in MSTO-211H **(B,C)**, REN **(D,E)** and MeT-5A cells **(F,G)**, that were serum deprived for 12 h and either untreated (c, control) or treated for 24 h with calcitriol (Cal), at the concentrations indicated. Blots are representative of three independent experiments. Results, normalized to actin and PARP for cytoplasmic or nuclear VDR, respectively, are expressed as percent of control and are means ± SEM. *P < 0.05, **P < 0.01, ***P < 0.001 vs. c.; *n* = 3.

To understand the role of calcitriol in MPM cell lines, we first assessed the presence of VDR. Western blot analysis revealed VDR protein expression in pleural biphasic MSTO-211H and epithelioid REN MPM cell lines, as well as in MeT-5A pleural mesothelial cells. The levels of VDR were significantly higher in MPM cells, particularly in REN cells, compared with mesothelial cells, where the receptor was barely detectable (Figure 1A). In addition, both cytoplasmic and nuclear VDR were increased in MSTO-211H (Figures 1B,C) and REN cells (Figures 1D,E) treated for 24 h with 50 and 100 nM calcitriol, compared with cells untreated. Conversely, no variation was observed in MeT-5A mesothelial cells, either in the absence or presence of calcitriol (Figures 1F,G). These results suggest that VDR expression is higher in MPM cell lines and is further induced by treatment with its ligand.

### Calcitriol Inhibits Viability and Proliferation in MPM Cell Lines

**Figure 2:**
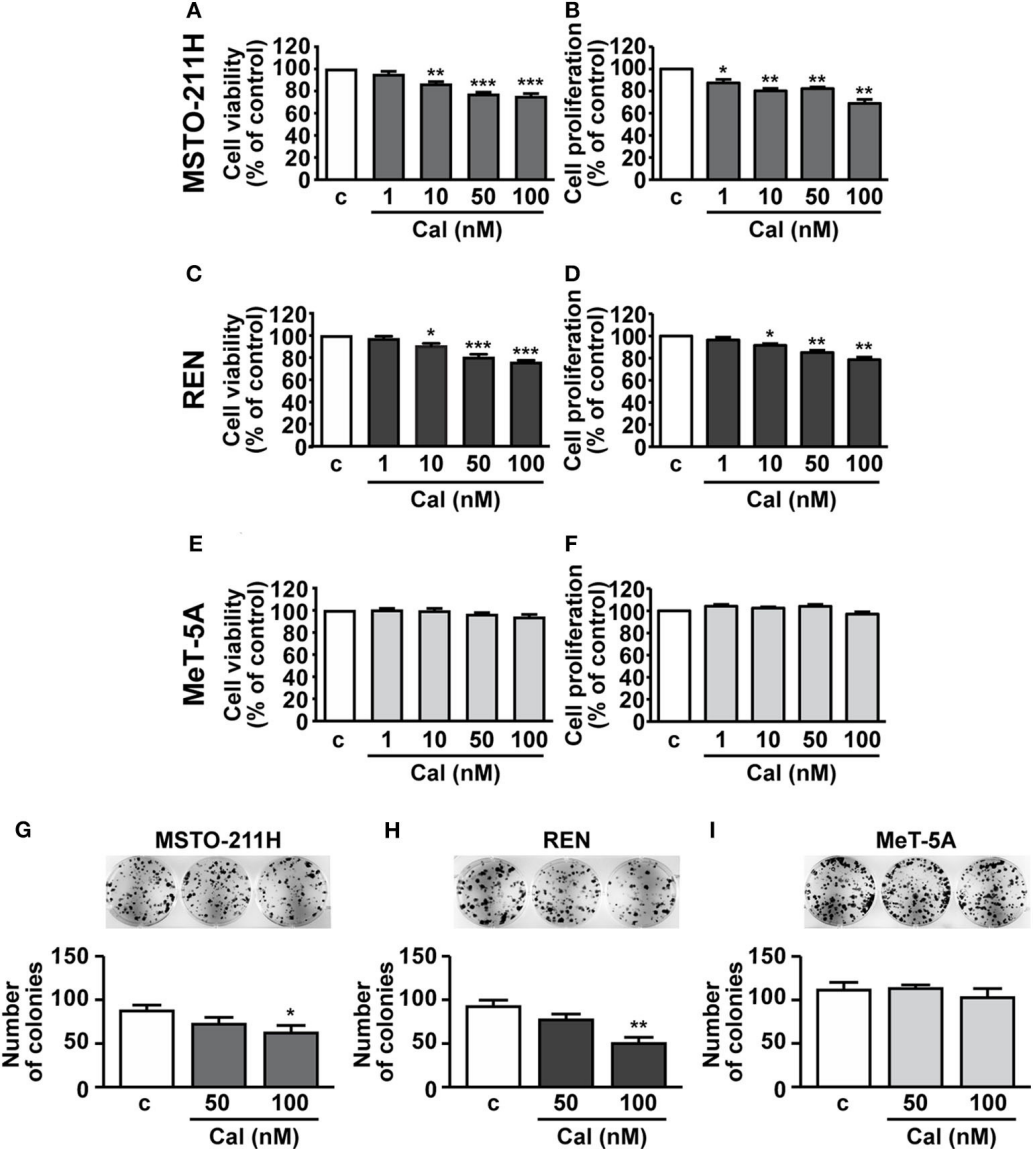
Effect of calcitriol on viability and proliferation of MPM cells and pleural mesothelial cells. MSTO-211H and REN MPM cells, and MeT-5A mesothelial cells, were serum deprived for 12 h, then cultured for 24 h in the absence (c, control) or presence of calcitriol (Cal), at the concentrations indicated. Cell viability **(A,C,E)** and proliferation **(B,D,F)** were assessed by MTT and BrdU, respectively. Maximum inhibition at 100 nM Cal in MSTO-211H **(A,B)**: 24.7 and 31.1%; REN: 24 and 21.3%, for viability and proliferation, respectively. Results, expressed as percent of control are means ± SEM. *P < 0.05, **P < 0.01, ***P < 0.001 vs. c; *n* = 8 for MTT, *n* = 4 for BrdU. Representative colony formation in MSTO-211H **(G)**, REN **(H)**, and MeT-5A cells **(I)** untreated (c, control) or treated for 10 d with calcitriol (Cal), at the concentrations indicated. Results are means ± SEM. *P < 0.05 and **P < 0.01 vs. c; *n* = 3.

We next assessed the potential inhibitory effects of calcitriol in MPM cells. As previously reported, (22), cell viability and proliferation were reduced of ~25% in MSTO-211H cells, ~20–25% in REN cells and ~15–18% in Met-5A cells (data not shown). Treatment with increasing concentrations of calcitriol (1 to 100 nM) for 24 further produced a dose-dependent reduction in viability and proliferation of both MSTO-211H (Figures 2A,B) and REN cells cultured in serum deprived medium (Figures 2C,D), compared with control. The maximum effect was observed at 50 nM (MSTO-211H: 22.6 and 17.8%; REN: 19.5 and 18% for viability and proliferation, respectively); and 100 nM (MSTO-211H: 24.7 and 31.1%; REN: 24 and 21.3% for viability and proliferation, respectively). By contrast, viability and proliferation were unchanged in non-malignant MeT-5A mesothelial cells treated with calcitriol, at any of the concentrations tested (Figures 2E,F). Similar findings were obtained for cell viability when the cells were cultured for 24 and 72 h in the presence of 2.5% serum, although the inhibitory effect of calcitriol was attenuated (100 nM calcitriol at 24 h, MSTO-211H: 12.8%; REN: 14.9%. 100 nM calcitriol at 72 h, MSTO-211H: 21.1%; REN: 23.1%) (Supplementary Figure 1). These findings suggest that calcitriol maintains its inhibitory activities even for longer-terms and in more physiological conditions. Because of the best inhibitory effect observed for both cell viability and proliferation, 100 nM was chosen as concentration for subsequent studies; in addition, due to the possible interference caused by serum, the experiments were performed under serum deprived medium. To further unveil the ability of calcitriol to reduce cell viability, we conducted the clonogenic assay and observed that after 10 days treatment, calcitriol blunted the ability of both MSTO-211H and REN cells to form colonies, while having no effect in MeT-5A mesothelial cells (Figures 2G–I).

### Calcitriol Potentiates the Inhibitory Effect of PEM in MPM Cell Lines

**Figure 3:**
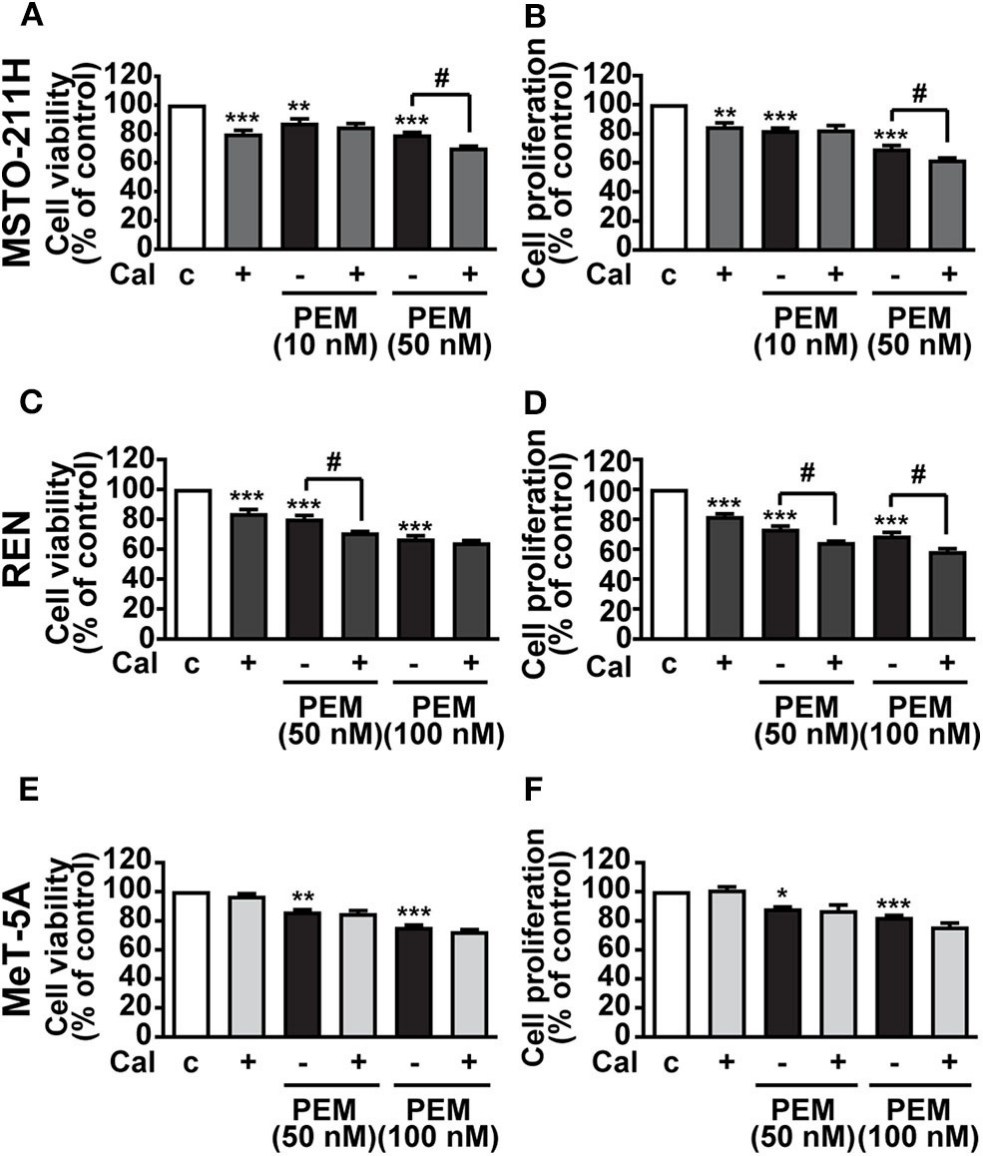
Inhibitory effects of calcitriol in combination with PEM. Cell viability and proliferation assessed by MTT and BrdU, respectively, in MSTO-211H **(A,B)** and REN **(C,D)** cell lines, and in MeT-5A mesothelial cells **(E,F)** treated for 72 h in medium with 2.5% serum (c, control) and with either 100 nM calcitriol (Cal) alone or with pemetrexed (PEM), at the concentrations indicated. Results, expressed as percent of control, are means ± SEM. *P < 0.05, **P < 0.01, ***P < 0.001 vs. c; ^#^*P* < 0.05, *n* = 4.

Having established its inhibitory function, we subsequently assessed whether calcitriol increases the cytotoxic effect of the anticancer drug PEM in MPM cells. MSTO-211H and REN cells, as well as MeT-5A mesothelial cells, were cultured in the presence of 2.5% serum and treated with calcitriol and PEM, either individually or in combination for 72 h, the appropriate time for PEM to be effective (22). PEM reduced cell viability and proliferation at 10 and 50 nM in MSTO-211H cells (Figures 3A,B) and at 50 and 100 nM in REN cells (Figures 3C,D), with the maximum inhibitory effect observed at the highest concentrations tested, as previously reported (22). In MSTO-211H cells, the most responsive to PEM, 100 nM calcitriol had no effect in combination with 10 nM PEM, while it potentiated the inhibitory activity of 50 nM PEM on both cell viability and survival (Figures 3A,B). The Combination Index (CI), calculated for viability in MSTO-211H cells, was <1 (0.944), indicating synergistic effect of 100 nM calcitriol and 50 nM PEM. In REN cells, the reduction induced by calcitriol was significant only with 50 nM PEM for cell viability (Figure 3C), likely because of the strong inhibitory effect of 100 nM PEM alone, with a CI < 1 (0.426), indicating synergistic effect of calcitriol and 50 nM PEM. Calcitriol also enhanced the effect of PEM 50 and 100 nM on reduction of cell proliferation (Figure 3D). Conversely, in line with the lack of effect observed previously in mesothelial cells (Figures 2E,F), calcitriol did not increase the cytotoxic activity of PEM in MeT-5A cells, at any of the concentrations tested (Figures 3E,F). Overall, these results indicate that calcitriol enhances the cytotoxic action of chemotherapy drugs in MPM cells, while having no effect in non-malignant cells.

### Calcitriol Promotes Cell Cycle Arrest and Inhibits the Expression of c-Myc and Cyclins in MPM Cells Lines

**Figure 4:**
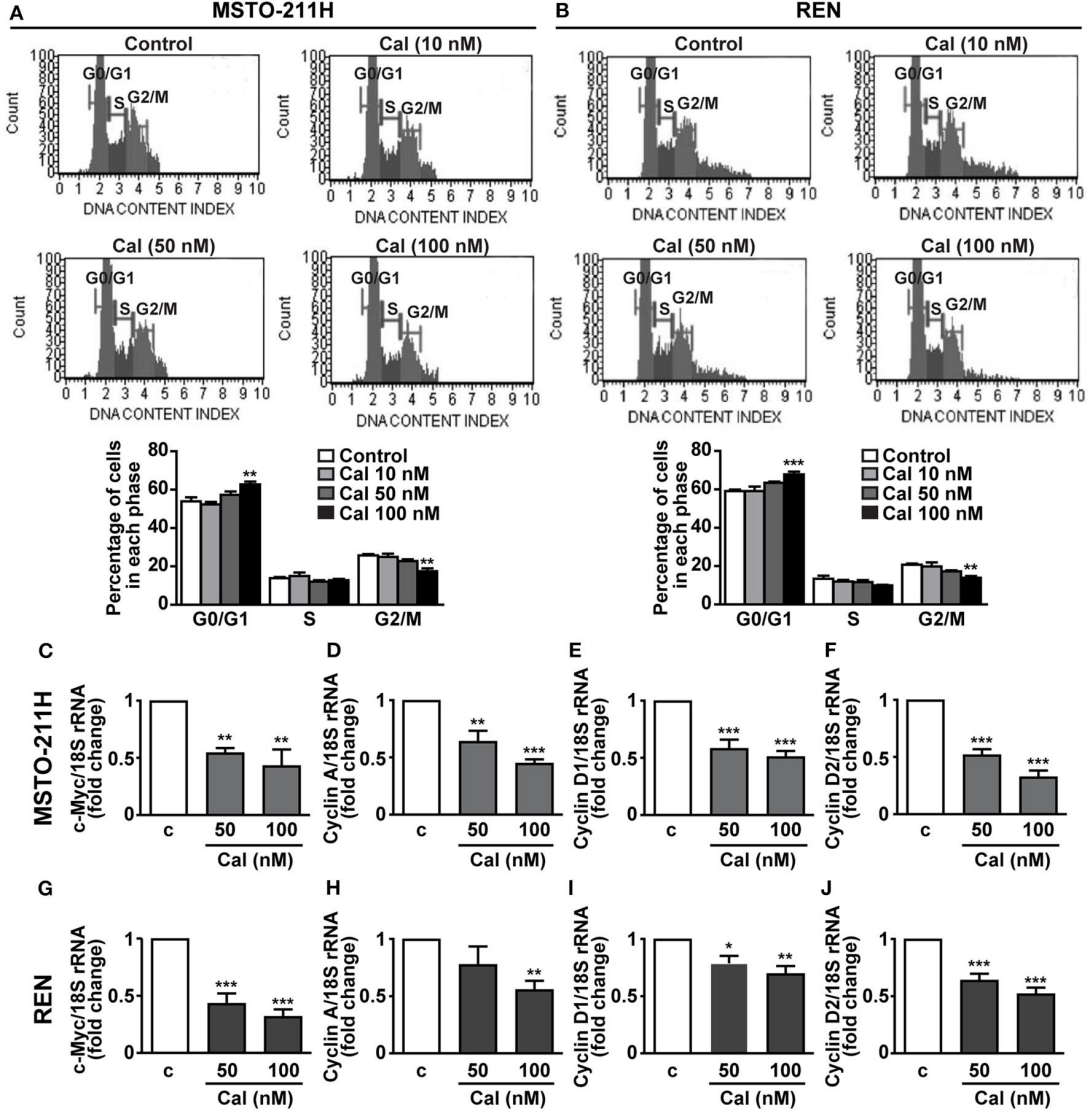
Role of calcitriol in cell cycle regulation in MPM cell lines. Representative images of cell distribution in G0/G1, S and G2/M phases analyzed by flow cytometry using the Muse Cell Cycle kit in MSTO-211H **(A)** and REN cells **(B)** (Top panels), cultured in the presence of 2.5% serum, and either untreated (Control) or treated with calcitriol (Cal) at the concentrations indicated. Results shown in graphs, are expressed as percentage of cells in each phase and are means ± SEM. **P < 0.01 and ***P < 0.001 vs. Control in each phase; n=3. Real-time PCR for c-Myc, cyclin A, cyclin D1, and cyclin D2 mRNA normalized to 18S rRNA in serum-starved MSTO-211H cells **(C–F)** and REN cells **(G–J)** treated for 24 h with calcitriol (Cal) at the concentrations indicated. Results, expressed as fold change of control (c), are means ± SEM of three independent experiments, each performed in duplicate. *P < 0.05, **P < 0.01 and ***P < 0.001 vs. c.

To investigate the mechanisms involved in the inhibitory activities of calcitriol, the cell cycle was analyzed in MPM cells stained with propidium iodide. In both MSTO-211H and REN cells, treatment with 100 nM calcitriol for 24 h caused an increase in accumulation of cells in the quiescent G0/G1 phase, in concomitance with a decrease in the proportion of cells in the active G2/M phase, while no effect was observed at the lowest concentrations tested (Figures 4A,B). In keeping with these findings, in MSTO-211H (Figures 4C–F) and REN cells (Figures 4G–L) we observed a reduced expression in the mRNA for *c-Myc* oncoprotein, and for *cyclin A, cyclin D1* and *cyclin D2*, all involved in cell cycle progression. By contrast, apoptosis was not affected in any of the cell lines tested (Supplementary Figures 2A–C). These findings suggest that the antitumor effects of calcitriol in MPM cells involve cell cycle arrest and inhibition of cell cycle progression.

### Calcitriol Reduces Mitochondrial Function in MPM Cell Lines

**Figure 5:**
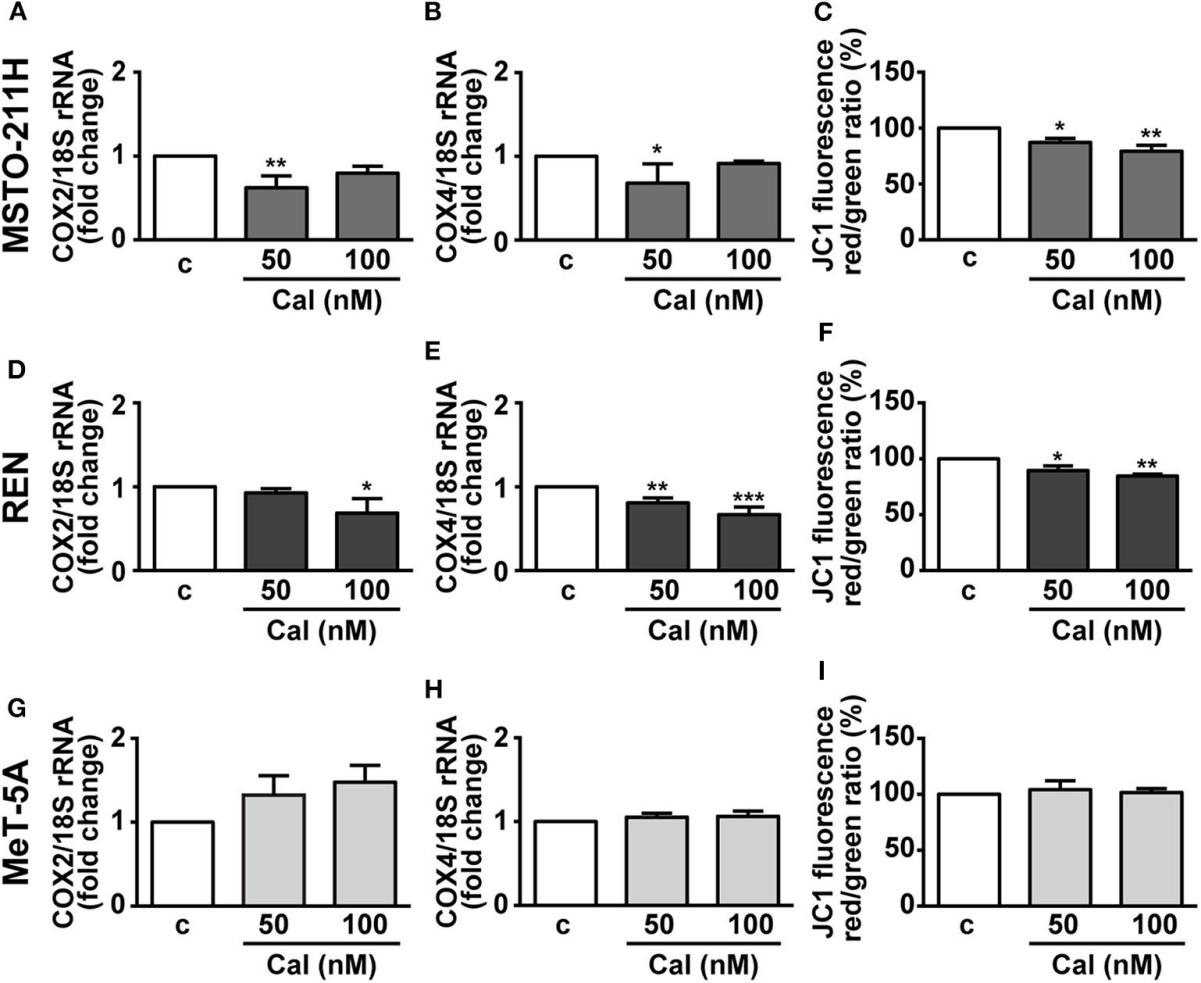
Effect of calcitriol on mitochondrial metabolism in MPM cells. Real-time PCR for *COX2* and *COX4* mRNA expression in MSTO-211H **(A,B)**, REN **(D,E)**, and MeT-5A cells **(G,H)**, serum starved for 12 h then treated for 24 h with calcitriol (Cal) at the concentrations indicated. Results, normalized to18S rRNA are expressed as fold change of vehicle are means ± SEM. *P < 0.05, **P < 0.01 and ***P < 0.001 vs. c; ns, not significant; *n* = 3. Mitochondrial membrane potential (ΔΨm) assessed by flow cytometry analysis of JC-1 in MSTO-211H **(C)**, REN **(F)** and MeT-5A cells **(I)** treated for 24 h in medium with 1% serum (c, control medium), and either with or without calcitriol (Cal) at the concentrations indicated. Red to green (FL-2/FL-1) ratio was calculated in each experimental condition and expressed as percent of untreated cells. Results are means ± SEM. *P < 0.05, *P < 0.01 vs. c; *n* = 3.

Mitochondria are essential regulators of cell viability, and cell death is classically preceded by mitochondrial alterations in the modulation of the complexes of respiratory chain and oxidative phosphorylation (OXPHOS), as well as the loss of mitochondrial membrane potential (ΔΨm) (27). In addition, previous studies have demonstrated the inhibitory effects of vitamin D on mitochondrial respiratory activity in both normal and cancer cells (16–18). Thus, mitochondrial function was first evaluated by analyzing the alterations in expression of two subunits of the respiratory chain complex IV, cytochrome c oxidase (COX) subunits 2 and 4, of mitochondrial and nuclear origin, respectively. Figures 5A,D shows that 24 h treatment with calcitriol reduced the mRNA expression of *COX2* at 50 nM in MSTO-211 and at 100 nM in REN cells. Similarly, we observed inhibition in *COX4* using 50 nM calcitriol in MSTO-211H cells, and with both the concentrations tested in REN cells (Figures 5B,E), whereas no changes were found for both subunits in MeT-5A cells (Figures 5G,H). Calcitriol also blunted ΔΨm in MSTO-211H (Figure 5C) and REN (Figure 5F) cells, while having no effect in MeT-5A cells (Figure 5I). Overall, these results suggest inhibitory activities for calcitriol in mitochondrial function in MPM cell lines, but not in non-malignant mesothelial cells.

### Calcitriol Decreases Cell Viability in Human Primary MPM Cells

**Figure 6:**
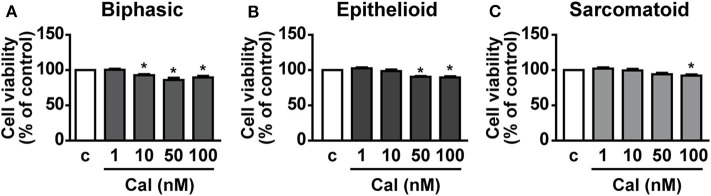
Inhibitory effects of calcitriol in primary MPM cells. Cell viability (MTT assay) in biphasic **(A)**, epithelioid **(B)**, and sarcomatoid **(C)** primary MPM cells cultured in serum-deprived medium (c, control) for 12 h, then treated for 24 h with calcitriol (Cal), at the concentrations indicated. Results, expressed as percent of control, are means ± SEM. *P < 0.05 vs. c; *n* = 3.

The effect of calcitriol was next assessed on viability of cells isolated from pleural biopsies of patients with MPM. Calcitriol was tested in a range of concentrations from 1 to 100 nM. In line with previous findings (22), cell viability was reduced of ~15–20% in biphasic, epithelioid, and sarcomatoid cells cultured in serum deprived conditions compared to the presence of serum (data not shown). The addition of calcitriol, at 10 to 100 nM in biphasic cells (Figure 6A), 50 and 100 nM in epithelioid cells (Figure 6B) and 100 nM in sarcomatoid cells (Figure 6C) caused a further decrease in cell viability, compared to untreated cells, suggesting antitumor activities also in human primary MPM cells.

## Discussion

This study shows that calcitriol, the active form of vitamin D, reduces cell viability and growth of MPM cells; furthermore, calcitriol potentiates the cytotoxic effect of the chemotherapy drug PEM. The anticancer activities of calcitriol, also observed in human primary MPM cells, involved inhibition of cell cycle progression and dysregulation of mitochondrial function.

The biological activity of calcitriol is mainly regulated by VDR, which modulates the expression of many target genes (5, 28). Importantly, it has been shown that tumor progression may be correlated with lower VDR levels in different types of cancer (5–9), and a positive association between expression of VDR/calcitriol with improved prognosis was found in patients with lung adenocarcinoma (29, 30). Furthermore, it has been recently demonstrated that vitamin D deficiency exacerbates pulmonary fibrosis in mice (31), whereas lung cancer cells expressing VDR are more responsive to the antiproliferative effect of calcitriol; in addition, high nuclear VDR expression has been associated with increased survival in lung cancer patients (29, 30, 32). Interestingly, studies suggested that an alteration in VDR/vitamin D_3_ axis in mouse alveolar macrophages may lead to pulmonary emphysema (33). In line with the above-mentioned findings, future studies should be undertaken to assess the levels of calcitriol and VDR in patients with MPM, which could have a relevant prognostic value.

Calcitriol was previously found to increase VDR expression in different cancer cells, as well as in bronchial epithelial cells and peritoneal mesothelial cells, along with inhibition of epithelial to mesenchymal transition (5, 8, 34). However, the presence of VDR in MPM cell lines has yet to be examined. Here, we show that VDR expression is much higher in MSTO-211H and REN than in MeT-5A cells, and is further increased by treatment with calcitriol, at both the cytoplasmic and nuclear level. VDR can be up-regulated by vitamin D at both the transcriptional and post-translational levels, the latter through a ligand-dependent stabilization (28). Possibly depending on both genomic (transcriptional) (35) or non-genomic (stabilization) (36) mechanisms, equally active after 24 h of treatment, we found that in MPM cell lines calcitriol increased VDR levels and promoted its nuclear translocation. Instead, basal VDR levels were modest in MeT-5A cells and remained unchanged after treatment with calcitriol, which would explain the lack of inhibitory activity in non-malignant cells, in line with previous findings (34). The induction of the catabolic enzyme 24-hydroxylase may justify the resistance of MeT-5A cells to vitamin D action, and possible differences among cell types, already described in other models (19), could be discovered also in mesothelial cells in future studies.

Another crucial difference among the cell lines tested could be due to the epigenetic remodeling affecting the vitamin D response elements (VDREs) present in many genes. In fact, VDREs mediate the genomic activity of vitamin D/VDR and control the transcription of VDR itself. The accessibility of VDREs is regulated by epigenetic mechanisms and modulated depending on the cell type and differentiation status (37). Alterations of VDR cistrome (the ensemble of VDREs open to VDR docking) could be responsible for different genomic responses to calcitriol in our experimental model; this is an interesting aspect that warrants further investigation.

The antitumor role of calcitriol and vitamin D derivatives in different types of cancer was demonstrated in several *in vitro* and *in vivo* studies, including lung cancer, suggesting that vitamin D may be a promising agent, in combination with anticancer therapies (5, 7–9, 38). Importantly, inflammation is one of the main hallmarks of cancer (39) and different studies have demonstrated the ability of vitamin D to modulate the innate and adaptive immune responses and to exert antinflammatory effects in tumorigenesis, by interfering with the tumor microenvironment through the regulation of key inflammatory pathways (5, 40, 41). Here, we report for the first time that calcitriol inhibits cell viability and growth of human epithelioid and biphasic MPM cell lines and blunts the viability of all primary MPM cell subtypes, suggesting anticancer properties in both the most and least aggressive phenotypes, while having no effect in mesothelial cells. These activities were observed at concentrations consistent with those previously reported in other cancer cell types (30, 42, 43). Interestingly, the ability of calcitriol to reduce viability in MPM cell lines was maintained even in more physiological conditions, i.e., in cells cultured with serum and after longer term treatment.

Robinson et al. previously showed that diet supplementation with cholecalciferol in a transgenic mouse model of asbestos-induced mesothelioma (MexTAg) had no effect on the incidence or severity of peritoneal mesothelioma (20). However, this model is different from the traditional human cancer xenograft (5, 8, 21); furthermore, mesothelioma cells from MexTAg mice showed increased expression of genes involved in the control of cell cycle, proliferation pathways or DNA replication, compared with those obtained from MPM of wild-type mice (44), likely because of the high expression of SV40 large T Antigen (TAg) in the mesothelial compartment, suggesting increased resistance to anticancer drugs. In addition, the direct action of cholecalciferol in mesothelioma cells of these mice was not examined (20).

Although the antitumor effects of calcitriol in preclinical models has been largely demonstrated, the single vitamin D analogs have limited efficacy in cancer therapy; thus, combination therapies with calcitriol and compounds such as glucocorticoids, cytotoxic drugs, inhibitors or retinoids have been widely investigated (9). Herein, we show that, in addition to the effect *per se*, calcitriol also acted in a synergistic manner with the antifolate compound PEM, which, in association with cisplatin, is currently the standard first-line treatment for patients with advanced MPM (4). To the best of our knowledge, this is the first evidence on the effective combination of calcitriol and PEM in cancer cells, despite vitamin D derivatives previously showed the ability to potentiate *in vitro* and *in vivo* the antitumor effects of platinum analogs like carboplatin and cisplatin (5, 9, 45). Therefore, although not suitable as a single use against cancer, vitamin D derivatives might be considered as adjuvants in combination therapy with cytotoxic compounds, as already described for other types of cancers (9, 11). On the other hand, vitamin E isoforms with anticancer properties were found to attenuate *in vitro* chemoresistance to cisplatin and to inhibit the growth of human MPM cell lines, without altering the growth of non-malignant mesothelial cells (46, 47).

In the present study, we did not observe an increase in apoptosis in MPM cells treated with calcitriol, in agreement with previous studies showing that calcitriol inhibits cell proliferation in human lung cancer and adrenocortical carcinoma cell lines, with no effect on apoptosis (30, 48). Interestingly, we found that calcitriol increases the proportion of cells in the quiescent phase of cell cycle (G_0_/G_1_), while reducing those in G2/M, indicating that the antiproliferative effects are associated with cell cycle arrest, in line with results in other cancer cells (8, 38). Furthermore, calcitriol inhibited the expression of *c-Myc* and *cyclin A, D1* and *D2*, all involved in the control of cell cycle (22, 24, 30, 49), in keeping with its ability to suppress the expression of c-Myc-regulated genes and to promote cell cycle arrest and inhibition of cyclins in both normal and cancer cells (30, 50, 51). c-Myc is a protooncogene frequently deregulated in many human cancers, whose inhibition induces apoptosis and sensitizes MPM cells to drug-induced cytotoxicity (52); moreover, we recently showed that c-Myc is implicated in the antitumor effects of growth-hormone releasing hormone (GHRH) antagonists in MPM cell lines (22).

In addition to the previously known nuclear effects of calcitriol in the control of cell proliferation, a novel finding of this study is the demonstration of the mitochondrial activity of VDR in mesothelioma. We and others previously demonstrated the ability of vitamin D to reduce the mitochondrial respiratory activity in both normal and cancer cells (16–18). Herein, calcitriol decreased the mitochondrial function, measured as inhibited transcription of respiratory complexes *COX2* and *COX4*, and as reduced membrane potential, which is proportional to the respiratory activity. Although the reduction of *COX2* and *COX4* was small, our result is in line with the fact that strong inhibition would lead to mitochondrial dysfunction and apoptosis (53), and we did not observed apoptosis after treatment of MPM cells with calcitriol, suggesting that the effects on *COX* expression are mostly metabolic. The consequences of a reduced mitochondrial activity can be different, depending on the sensitivity to the differentiating nuclear activity of vitamin D and according to the genetic and epigenetic asset of the cells, as demonstrated in our studies in human keratinocytes and cancer cell lines (17–19). When the cells are resistant to the nuclear differentiating effects, the reduced mitochondrial activity reroutes the metabolic intermediates toward the biosynthetic pathways supporting proliferation (17), whereas when vitamin D exerts its nuclear control, the spared intermediates are employed in the specific metabolism of the tissue (19). In human MPM cells we found that calcitriol exerts nuclear antiproliferative effects and potentiates the antiproliferative activity of PEM in a synergistic manner. In addition, the inhibition of mitochondrial activity must reduce the energy supply of mesothelioma, thus increasing the cytotoxicity of the drug, possibly by a reduced ATP-dependent drug extrusion. Similarly, we recently reported the reduction of ΔΨm in response to the antiproliferative effects of GHRH antagonists in MPM cell lines (21). Differently from other studies that demonstrated an association between cell growth arrest and apoptosis, due to the inhibition of OXPHOS complex subunits and ΔΨm in MPM treated with mitochondria-targeted molecules (43), we did not find evidences for a proapoptotic effect of mitochondrial inhibition.

Overall, the results of this study demonstrate the inhibitory effect of calcitriol on viability and growth of MPM cells, either alone or in combination with PEM, through mechanisms involving cell cycle arrest and inhibition of the mitochondrial activity, suggesting that vitamin D derivatives, in association with chemotherapy drugs, could have potential in the treatment of MPM.

## Data Availability Statement

The raw data supporting the conclusions of this article will be made available by the authors, without undue reservation.

## Ethics Statement

The studies involving human participants were reviewed and approved by the Ethical Committees of the Biological Bank of Mesothelioma, SS. Antonio and Biagio General Hospital, Alessandria, Italy, and San Luigi Gonzaga Hospital, Orbassano, Turin, Italy (#9/11/2011; #126/2016). The patients/participants provided their written informed consent to participate in this study.

## Author Contributions

IG, FS, DB, VM, AF, GG, and NC performed research, organized the database, and performed the statistical analysis. CR and RL provided the primary MPM cells. EG and RG concepted the research RG, IG, and FS designed the research. RG wrote the paper. All authors contributed to the revision of the manuscript and approved the submitted version.

## Conflict of Interest

The authors declare that the research was conducted in the absence of any commercial or financial relationships that could be construed as a potential conflict of interest.
