# Supplementary material for: Calcitriol Inhibits Viability and Proliferation in Human Malignant Pleural Mesothelioma Cells
Source: Front Endocrinol (Lausanne). 2020 Oct 8;11:559586. doi: 10.3389/fendo.2020.559586 (PMC7579995; doi:10.3389/fendo.2020.559586)
Supplement: Supplementary file 1 [file Data_Sheet_1.PDF]

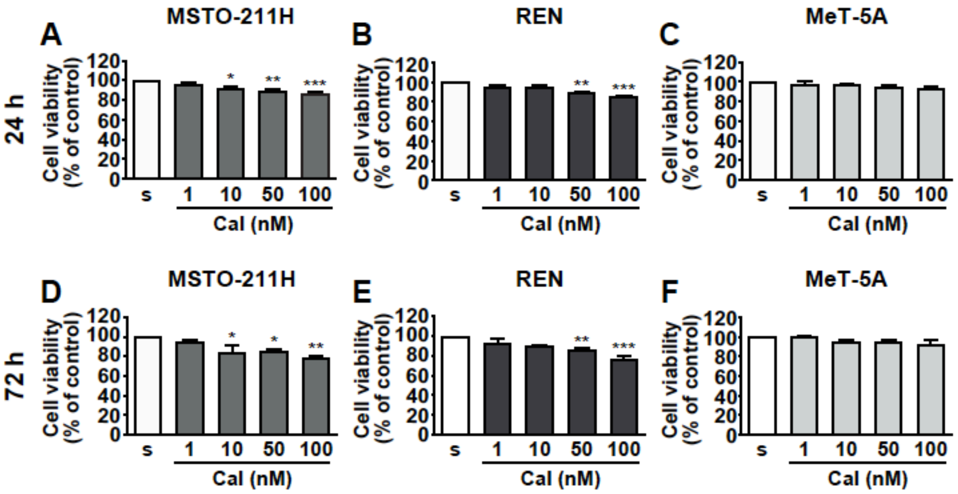

Supplementary Figure 1. Effect of calcitriol on viability of MPM cells and pleural mesothelial cells cultured in the presence of serum. Cell viability was assessed by MTT in MSTO-211H, REN and MeT-5A treated with 100 nM calcitriol (Cal) in the presence of 2.5% serum (s), at either 24 h (A-C) or 72 h (D-F). Results, expressed as percent of control, are means ± SEM. *$P < 0.05$, **$P < 0.01$, ***$P < 0.001$ vs. s; n=3.

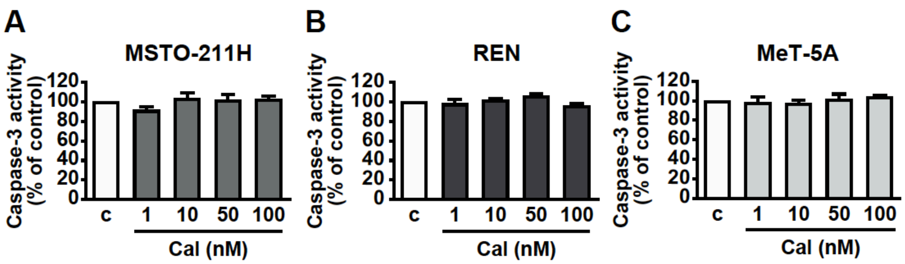

Supplementary Figure 2. Caspase-3 activity in MSTO-211H (A), REN (B) and MeT-5A cells (C) treated for 24 h with calcitriol (Cal) at the concentrations indicated. Results, expressed as percent of control are means ± SEM. n = 3.
